# Supplementary material for: Combining ability, heterosis and performance of grain yield and content of Fe, Zn and protein in bread wheat under normal and late sowing conditions
Source: PeerJ. 2023 Mar 9;11:e14971. doi: 10.7717/peerj.14971 (PMC10008312; doi:10.7717/peerj.14971)
Supplement: Supplemental Information 1 [file peerj-11-14971-s001.docx]

| **Male Parents** | | | | | | | | | | | |
| --- | --- | --- | --- | --- | --- | --- | --- | --- | --- | --- | --- |
| **Female Parents** |  | P1 | P2 | P3 | P4 | P5 | P6 | P7 | P8 | P9 | P10 |
|  | P1 | GW 451 | P1× P2 | P1 × P3 | P1 × P4 | P1 × P5 | P1 × P6 | P1 × P7 | P1 × P8 | P1 × P9 | P1 × P10 |
|  | P2 |  | GW 496 | P2 × P3 | P2 × P4 | P2 × P5 | P2 × P6 | P2 × P7 | P2 × P8 | P2 × P9 | P2 × P10 |
|  | P3 |  |  | LOK 1 | P3 × P4 | P3 × P5 | P3 × P6 | P3 × P7 | P3 × P8 | P3 × P9 | P3 × P10 |
|  | P4 |  |  |  | GW 322 | P4 × P5 | P4 × P6 | P4 × P7 | P4 × P8 | P4 × P9 | P4 × P10 |
|  | P5 |  |  |  |  | GW 366 | P5 × P6 | P5 × P7 | P5 × P8 | P5 × P9 | P5 × P10 |
|  | P6 |  |  |  |  |  | HI 1544 | P6 × P7 | P6 × P8 | P6 × P9 | P6 × P10 |
|  | P7 |  |  |  |  |  |  | GW 173 | P7 × P8 | P7 × P9 | P7 × P10 |
|  | P8 |  |  |  |  |  |  |  | GW 11 | P8 × P9 | P8 × P10 |
|  | P9 |  |  |  |  |  |  |  |  | HD 2864 | P9 × P10 |
|  | P10 |  |  |  |  |  |  |  |  |  | UAS 385 |
| **Standard check varieties** | 1. | MACS 6222 – TS | | | | | | | | | |
|  | 2. | HD 2932 – LS | | | | | | | | | |

**Supplementary Table 1** Crossing pattern in 10 × 10 half diallel crosses in normal and late environments (*Rabi-2017-18*)**.**

| **Sr. No.** | **Released varieties** | **Pedigree** | **Source** |
| --- | --- | --- | --- |
| 1 | GW 451 | GW324/4/CROC_1/AE.SQUARROSA (205) /JUP/ JY/3/ SKAUZ /4/ KAUZ /5/ GW 339 | Centre of Excellence for  Research on Wheat,  SDAU, Vijapur 382 870 |
| 2 | GW 496 | HD 2285/CPAN 1861 |  |
| 3 | LOK 1 | S 308/S 311 |  |
| 4 | GW 322 | PBW 173/GW 196 |  |
| 5 | GW 366 | DL 802-3/GW 232 |  |
| 6 | HI 1544 | HIND162/BOBWHITE/CPAN 2099 |  |
| 7 | GW 173 | TW 275 -7-6-10/LOK1 |  |
| 8 | GW 11 | LOK 1 / HW 1042//LOK 1 |  |
| 9 | HD 2864 | DL509-2/DL377-8 |  |
| 10 | UAS 385 | GW344/UAS239/DWR162 |  |
| **Standard check varieties** | | |  |
| 1 | MACS 6222 – TS  (SH1) | HD 2189*2//MASC 2496 |  |
| 2 | HD 2932 – LS  (SH2) | KAUZ/STAR//HD 2643 |  |

**Supplementary Table 2** List of genotypes their pedigree information and sources

| **Time of application** | **N (Kg/ha)** | **P (Kg/ha)** | **K (Kg/ha)** |  | **Irrigation** |
| --- | --- | --- | --- | --- | --- |
| E_1_ (Normal) Basal dose + Splits as usual | 120 | 60 | 00 |  | Post sowing surface irrigation and irrigation as scheduled. |
|  | 60 | 60 | 00 | Basal dose |  |
|  | 60 | 00 | 00 | Split dose |  |
| E_2_ (Late) Basal dose + One split | 90 | 60 | 00 |  |  |
|  | 45 | 60 | 00 | Basal dose |  |
|  | 45 | 00 | 00 | Split dose |  |

Note: Splits as usual

**Supplementary Table 3** Nutrient management and irrigation in wheat at Regional Research Station, AAU, Anand

|  | **Normal** | | | | **Late** | | | |
| --- | --- | --- | --- | --- | --- | --- | --- | --- |
|  | **GYP** | **PC** | **FE** | **ZN** | **GYP** | **PC** | **FE** | **ZN** |
| **GSCA versus** |  |  |  |  |  |  |  |  |
| **GCA** | 0.80** | 0.77** | 0.66** | 0.67** | 0.66** | 0.65** | 0.86** | 0.61** |
| **SCA** | 0.24 | -0.05 | -0.08 | 0.08 | 0.06 | 0.12 | 0.11 | 0.26 |
| **BPH** | 0.45** | -0.07 | -0.20 | 0.05 | 0.24 | 0.13 | 0.23 | 0.61** |
| **SH1** | 0.72** | 0.53** | 0.26 | 0.60** | 0.41** | 0.55** | 0.39** | 0.69** |
| **SH2** | 0.72** | 0.53** | 0.26 | 0.60** | 0.41** | 0.55** | 0.39** | 0.69** |
| **Phenotype** | 0.72** | 0.52** | 0.25 | 0.60** | 0.41** | 0.55** | 0.39** | 0.69** |
| **GCA versus** |  |  |  |  |  |  |  |  |
| **SCA** | 0.16 | 0.11 | -0.24 | 0.13 | 0.17 | 0.11 | 0.13 | 0.44** |
| **BPH** | 0.41** | 0.13 | -0.24 | 0.16 | 0.23 | 0.20 | 0.29* | 0.56** |
| **SH1** | 0.55** | 0.55** | 0.001 | 0.47** | 0.39** | 0.39** | 0.37* | 0.63** |
| **SH2** | 0.55** | 0.55** | 0.001 | 0.47** | 0.39** | 0.39** | 0.37* | 0.63** |
| **Phenotype** | 0.55** | 0.54** | 0.0003 | 0.47** | 0.39** | 0.37* | 0.37* | 0.63** |
| **SCA versus** |  |  |  |  |  |  |  |  |
| **BPH** | 0.85** | 0.82** | 0.80** | 0.90** | 0.86** | 0.84** | 0.82** | 0.85** |
| **SH1** | 0.85** | 0.81** | 0.92** | 0.84** | 0.93** | 0.89** | 0.95** | 0.88** |
| **SH2** | 0.85** | 0.81** | 0.92** | 0.84** | 0.93** | 0.89** | 0.95** | 0.88** |
| **Phenotype** | 0.85** | 0.81** | 0.92** | 0.84** | 0.93** | 0.88** | 0.95** | 0.87** |

**Supplementary Table 4** Estimation of relative GSCA (General Sum of Combining Ability) effects of crosses for grain yield and grain protein, Fe

and Zn concentration in normal and late condition. (*Rabi* 2018-19)

GSCA - General Sum of Combining Ability; GCA – General Combining Ability; SCA – Specific Combining Ability; SH1– Standard Heterosis 1; SH2– Standard Heterosis 2; BPH – Best-Parent Heterosis; GYP – Grain Yield Per Plant; PC – Grain Protein content FE – Grain Fe content; ZN – Grain Zn content. *P < 0.05; **P < 0.01; ***P < 0.001\
